# ﻿Three new monobasic genera and three new species of the New World treehopper tribe Acutalini (Hemiptera, Membracidae, Smiliinae) with a key to all genera

**DOI:** 10.3897/zookeys.1143.94124

**Published:** 2023-01-31

**Authors:** Stuart H. McKamey

**Affiliations:** 1 Systematic Entomology Laboratory, Agricultural Research Service, U.S. Department of Agriculture, c/o National Museum of Natural History, P.O. Box 37012, Washington, D.C. 20013, USA c/o National Museum of Natural History Washington, D.C. United States of America

**Keywords:** Brazil, Costa Rica, Ecuador, French Guiana, Neotropical, new genus, Peru

## Abstract

Three new genera in Acutalini are described, two of which have two discoidal cells (R_2+3_ and M) in the forewing, as in *Euritea* Stål. *Ceresinoideazacki***gen. nov. et sp. nov.**, from Guatemala, differs from other acutalines in having a pair of suprahumeral spines and a stepwise convex pronotum in lateral view. *Quinquespinosaseptamacula***gen. nov. et sp. nov.**, which is widely distributed in South America, differs in having a basal cell M and three posterior pronotal spines. *Tectiformaguayasensis***gen. nov. et sp. nov.**, from Ecuador, has the pronotum strongly tectiform throughout. A key to all genera of Acutalini is provided.

## ﻿Introduction

Acutalini belongs to the second most speciose treehopper subfamily, Smiliinae (Bartlett et al. 2018), with 750 species. Acutalini, however, is a species-poor tribe, with only 26 described species, but ranges from Canada to Brazil and Peru. At the time of Deitz’s (1975) revised classification of the New World Membracidae, Acutalini contained only three genera: *Acutalis* Fairmaire (nine species), *Euritea* Stål (three species), and *Thrasymedes* Kirkaldy (six species). All of these species were listed by McKamey (1998) and are elongate with a low, dorsally convex pronotum that lacks suprahumeral spines (McKamey 1998 also listed *Acutalisterminalis* Walker, which was designated as the type species of *Germariana*Sakakibara 1998). Acutaline genera differ from each other by forewing venation patterns, and differ from other Smiliinae in the following combination of characters: having the pronotum not, or only slightly, overlapping the forewing in repose, having veins R, M, and Cu separate near the wing base, vein R_2+3_ present as a distinct branch of R, vein R_4+5_ confluent with M distad of M fork, and crossveins s and m-cu present (Deitz 1975). Dietrich et al. (2001) also included, as another feature, a forked anal vein in the hind wing (as in Fig. 39), which is shared with non-smiliines but only two other tribes of Smiliinae (Ceresini and Micrutalini).

Sakakibara (1997) described another genus, *Bordonia* (preoccupied, replaced by *Bordoniana*Sakakibara 1999), with five species, and also the genus *Cornutalis*, with two species. Both genera have a pair of short, laterally directed suprahumeral spines. Flórez-V (2017) described another species of *Cornutalis* from Colombia that has a pitted pronotum with stout suprahumeral spines directed dorsoanteriorly.

In a recent sequence-based phylogenetic study (Evangelista et al. 2017), Micrutalini and Acutalini had intermixed clades and were, together, the sister group of Cymbomorphini, not with Ceresini or other Smiliinae. McKamey and Wallner (2022) described the immature stages of Acutalini and Micrutalini.

In the present paper, three new genera and three new species are described. Two of these new genera would follow Sakakibara’s (1997) key to *Euritea* because they have two discoidal cells in the forewing. Nevertheless, they differ from *Euritea* in important respects: they have suprahumeral spines or are strongly tectiform. A key is provided to all genera of Acutalini.

## ﻿Material and methods

In quoting labels, quotation marks separate labels and a vertical line separates lines on a label. Terminology for general morphology, forewing venation, and leg chaetotaxy follows Deitz (1975). A Leica MZ12 stereomicroscope was used to examine structures. The body length was measured using a digital micrometer. A manual 5 mm micrometer was used to determine ratios between other, shorter distances.

The abdomen was detached, macerated in a warmed 10% KOH solution for 24 hours at room temperature, bathed in water, then acetic acid to stop the reaction. After dissection, structures were stored in a glass microvial containing glycerin and pinned beneath the specimen.

Images were taken with a Canon 5D SLR camera with an adjustable 65 mm macro lens using Capture One Pro ver. 10.1.2, 64 Bit, aided by CamLift ver. 2.9.7.1. The specimens were lit using two adjustable Dynalite MH2050 RoadMax flash heads, each attached to a Manfrotto 244 arm. The light was diffused using a simple, lampshade-style cone of translucent paper between the specimen and light sources. After individual “slices” were photographed, they were compiled into a single, composite image using Zerene Stacker - USDA SI-SEL Lab Bk imaging system, ver. 1.04. Stacked images were enhanced and edited in Adobe Photoshop CSS Extended ver. 12.0. The scale bars were generated through Photoshop directly from the metadata of the photo.

Specimens examined will be deposited in the following Institutions:

**EPNC** Ecuador, Pichincha, Quito, Museo de la Escuela Politécnica Nacional;

**INPA**Brazil, Amazonas, Manaus, Instituto Nacional de Pesquisas da Amazonia, Colecão Sistemática da Entomologia;

**MNHN**France, Paris, Museum National d’Histoire Naturelle;

**MUSM**Peru, Lima, Universidad Nacional Mayor de San Marcos, Museo de Historia Natural;

**USNM**USA, U.S. National Museum of Natural History, Smithsonian Institution, Washington, DC;

**UVGC**Guatemala, Guatemala City, Universidad del Valle de Guatemala, Colección de Artópodos.

## ﻿Results

### ﻿Key to genera of Acutalini (modified from Sakakibara 1997)

**Table:** 

1	Forewing without discoidal cells (Fig. 1)	***Acutalis***
–	Forewing with 1 or 2 discoidal cells (Figs 2–4)	**2**
2	Forewing with 2 discoidal cells (R_2+3_ and M; Fig. 2)	**3**
–	Forewing with 1 discoidal cell (Figs 3, 4)	**5**
3	Pronotum with suprahumeral spines	***Ceresinoidea* gen. nov.**
–	Pronotum without suprahumeral spines	**4**
4	Pronotum dorsally convex without distinct median carina	***Euritea***
–	Pronotum strongly tectiform with distinct median carina	***Tectiforma* gen. nov.**
5	Forewing without basal cell M (Fig. 3)	***Thrasymedes***
–	Forewing with basal cell M (Figs 4, 21, 24)	**6**
6	Forewing with discoidal cell R_2+3_ (Fig. 21), but without discoidal cell M	***Quinquespinosa* gen. nov.**
–	Forewing without discoidal R_2+3_ but with discoidal cell M (Fig. 4)	**7**
7	Pronotum with pair of suprahumeral spines	***Cornutalis***
–	Pronotum without pair of suprahumeral spines	***Bordoniana***

#### ﻿ Ceresinoidea gen. nov.

Taxon classificationAnimaliaHemipteraMembracidae

7A23795C-FC34-50AD-AE99-684AC045DC11

https://zoobank.org/BBA05B12-D28D-465B-8077-2F59C37D8B59

Figs 5–11 , 12–17

##### Type species.

*Ceresinoideazacki* sp. nov.

**Figures 1–4.**
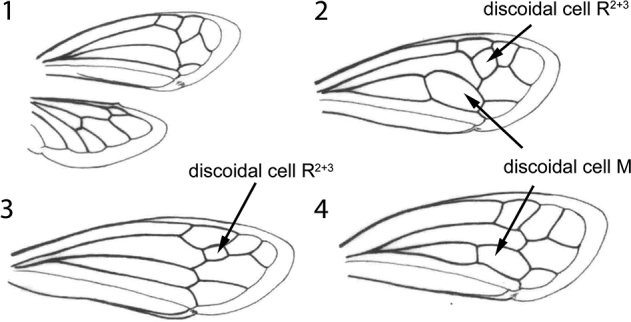
Wings of Acutalini genera **1** forewing and hind wing of *Acutalis***2** forewing of *Euritea***3** forewing of *Thrasymedes***4** forewing of *Bordoniana*. (Modified from Sakakibara 1997; licensed under a Creative Commons License, with permission of the editor).

##### Diagnosis.

**Figures 5–11.**
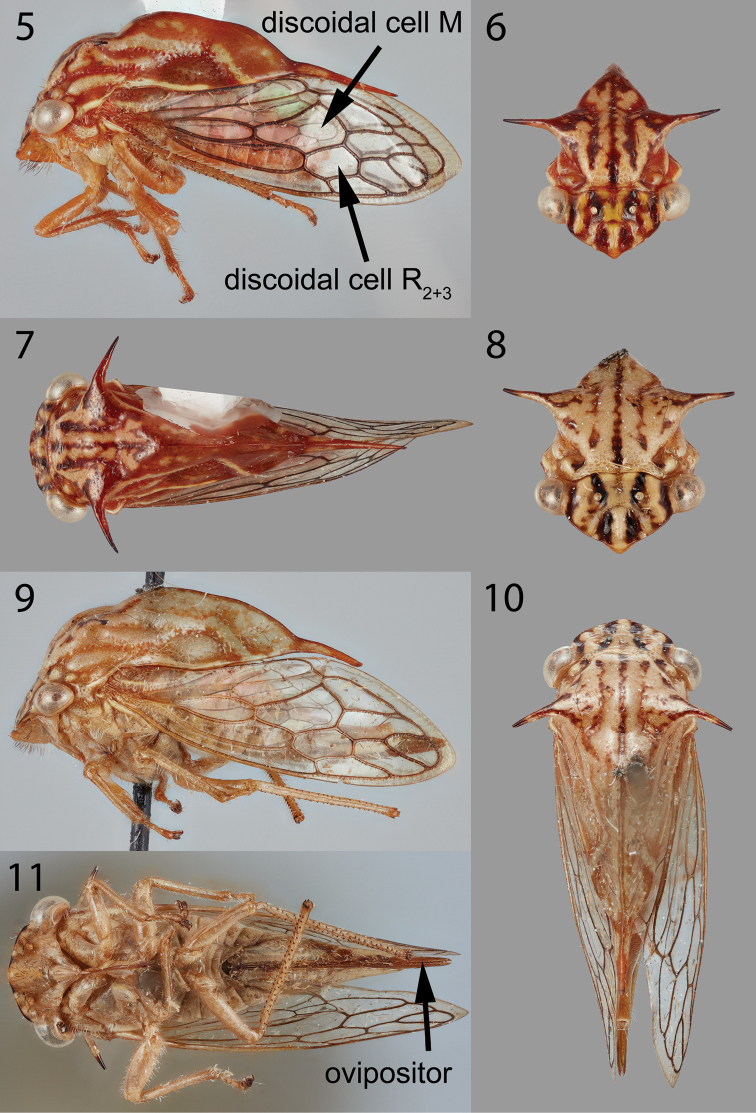
*Ceresinoideazacki*, sp. nov. **5–7** male habitus in lateral, anterior, and dorsal views, respectively **8–11** female habitus in anterior, lateral, dorsal, and ventral views, respectively.

Forewing with cells R_2+3_ and M, and 2 m-cu crossveins (Fig. 5); pronotum with suprahumeral spines directed laterally and slightly posteriorly (Figs 6–8, 10), distally attenuate posteriorly (Figs 5, 10).

##### Description.

***Head*.** Vertex glabrous, without ridges or rugae, slightly concave especially at lateral margins and around ocelli; ocelli circular, slightly closer to eyes than to each other; dorsal margin weakly convex but not attaining dorsal margin of eye, which is elevated (Figs 6, 8); frontoclypeus apically rounded, its sutures arched to mid-point. ***Pronotum*.** Smooth, glabrous, elevated, strongly convex in lateral view, with acute suprahumeral spines. ***Wings*.** Forewing (Figs 5, 9) with 2 adjacent discoidal cells (R_2+3_ and M), 2 m-cu crossveins. Hind wing with 1 r-m and 1 m-cu crossvein, with forked anal vein. ***Legs*.** Metathoracic tibia with cucullate setae in row I double, row II and row III complete and single.

##### Distribution.

Neotropical: Central America.

##### Etymology.

The name, which is feminine, is based on the superficial similarity of the type species to inornate members of the tribe Ceresini.

#### ﻿ Ceresinoidea zacki sp. nov.

Taxon classificationAnimaliaHemipteraMembracidae

B2BCA5DE-80CC-5327-8918-3331EC55B644

https://zoobank.org/9C5EA1ED-8968-474A-A1BB-8468D2AB1862

Figs 5–11 , 12–17

##### Diagnosis.

Pronotum elevated, stepwise convex just behind suprahumeral spines in lateral view (Fig. 5, 9), posteriorly strongly tectiform and compressed laterally, in anterior view with 3 irregular vertical stripes (Fig. 6, 8).

##### Description.

**Figures 12–17.**
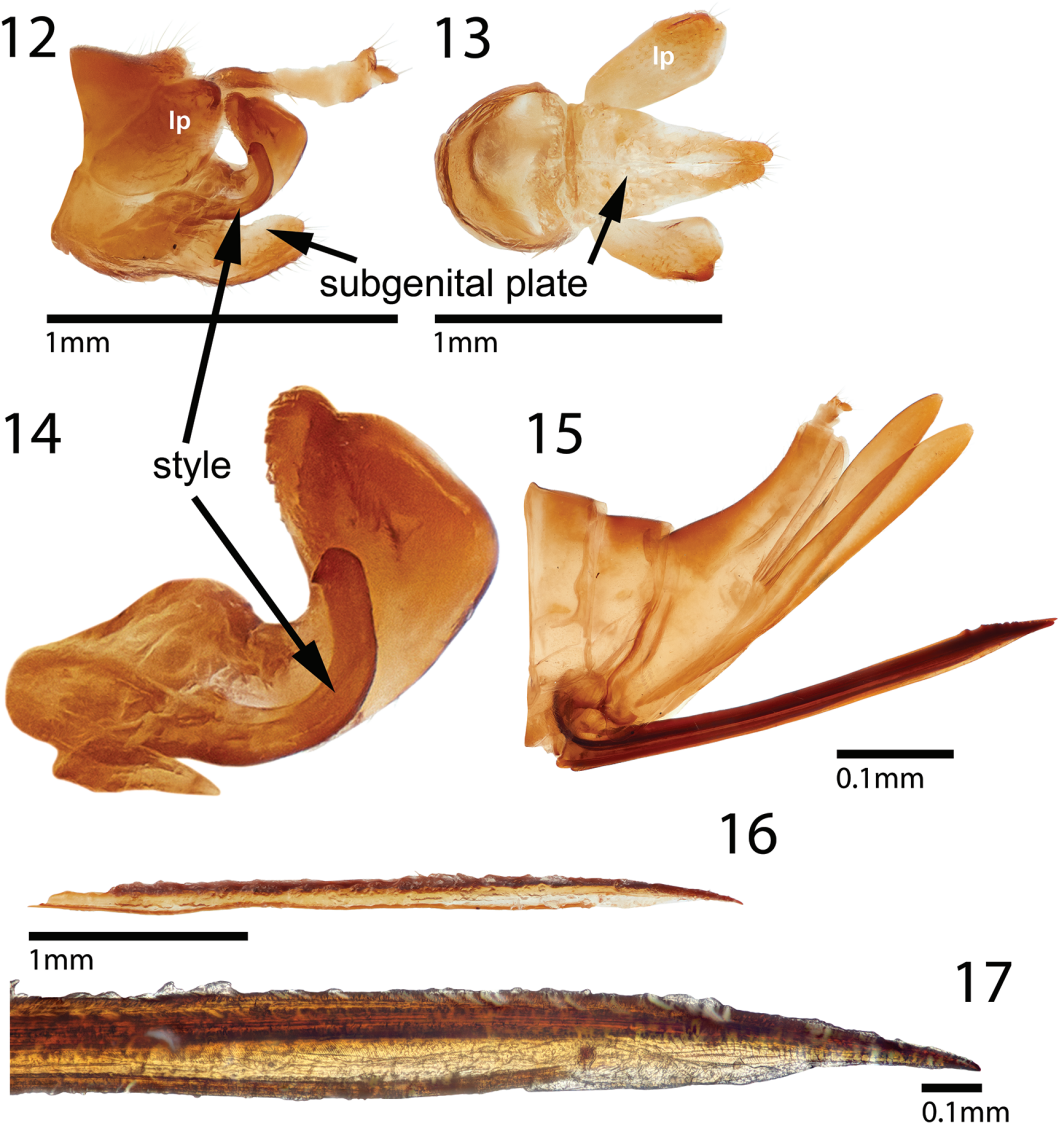
Terminalia of *Ceresinoideazacki*, sp. nov. **12** undissected terminalia of male **13** ventral view of subgenital plate and unarmed lateral plates **14** aedeagus and style, lateral view **15** undissected female terminalia **16, 17** second valvula (base broken) and distal portion, lateral view. *lp*, lateral plate.

Measurements (mm). Length with forewing in repose ♀ 8.6, ♂ 6.4; width across suprahumeral spines ♀ 3.7, ♂ 3.0; height in anterior view ♀ 3.0, ♂ 2.7. ***Pronotum*.** Dorsal margin abruptly elevated behind suprahumeral spines, convex in stepwise fashion (Figs 5, 9); suprahumeral spines narrow, directed laterally and slightly posteriorly, apices acute (Figs 6–8, 10); strongly tectiform posteriorly (Figs 7, 10). ***Terminalia*.** Male lateral plates unarmed (Figs 12, 13); subgenital plate triangular in ventral view, fused basally (Fig. 13); style recurved with short acute apex (Fig. 14); aedeagal shaft in lateral view thickest at mid-length, serrate along swollen anterodistal margin (Fig. 14). Female pygofer long, ovipositor extending even further (Figs 11, 15), together accounting for more than half of body length; second valvula simple, without dentae preapically (Figs 16, 17). ***Color*.** Female coloration (Figs 8–11): overall pale, vertex lateral margins black with two vertical black stripes passing over ocelli onto frontoclypeus; pronotum very pale brown with a darker brown central stripe terminating just behind suprahumeral spine, continuing as even paler stripe that arches ventrally to lateral margin, which is also very pale, two indistinct stripes on metopidium, and mottling from base to apex of suprahumeral spines. Wing veins and body pale brown. Male coloration (Figs 5–7) similar to female but all stripes darker, and areas except for stripes orange.

##### Material examined.

***Holotype*** ♂ (USNM) with labels “GUATEMALA: Peten Dept. Rio | Machaquila, ca. 8 km W of | Machaquila. 15–16 Aug 2015. | N16.39957° W89.48642° 413m, | light traps. R. S. Zack collector” and a red “HOLOTYPE | Ceresinoidea | zacki | S.H. McKamey. Slightly broken: right metathoracic leg missing. ***Paratype*** ♀ (UVGC)) with labels “GUATEMALA: Izabal Dept | Finca Firmeza, Reserva de | Anfibios, SE of Morales, 540m | N15.40689°, W88.69603° 3–4 | June 2016. R. S. Zack, light traps” and a blue paratype label. Both metathoracic tarsi missing, otherwise intact.

##### Distribution.

Guatemala.

##### Etymology.

The specific epithet is a patronym for Dr Richard Zack, who collected the holotype and paratype.

##### Note.

The holotype and paratype were collected at light traps, indicating a good method to discover more specimens. Among membracids, females are usually only slightly larger than males; in this species the female is significantly larger. The long ovipositor (Fig. 11) is also unusual among membracids.

#### ﻿ Quinquespinosa gen. nov.

Taxon classificationAnimaliaHemipteraMembracidae

2CCA2915-05EC-5966-B2AB-36CB47620707

https://zoobank.org/8CC5CAA6-7C49-4368-9374-063D758230B8

Figs 18–24 , 25–31

##### Type species.

*Quinquespinosaseptamacula* sp. nov.

##### Diagnosis.

Forewing with basal cell M and discoidal cell R_2+3_, without discoidal cell M, 1 m-cu crossvein; pronotum with 2 suprahumeral and 3 apical, slender spines.

##### Description of female.

**Figures 18–24.**
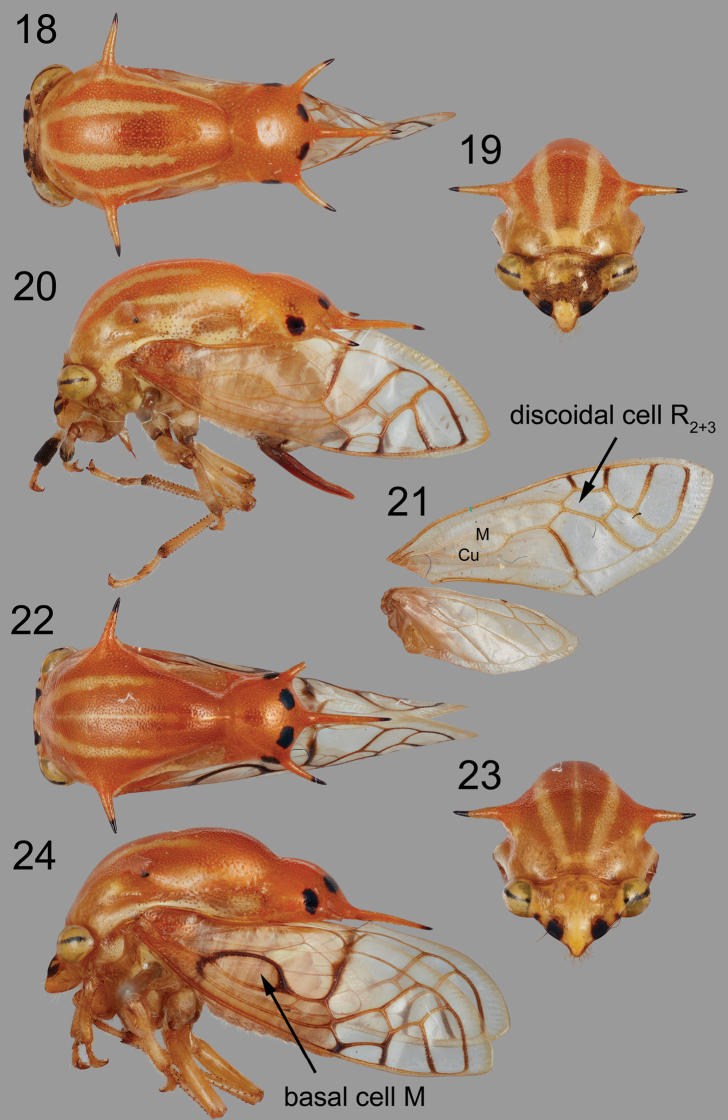
*Quinquespinosaseptamacula*, sp. nov. **18–20** female habitus in dorsal, anterior, and lateral views, respectively **21** forewing and hind wing **22–24** male habitus in dorsal, anterior, and lateral views, respectively.

***Head*.** Vertex glabrous, without ridges, slightly concave with linear furrow between ocellus and eye; ocelli slightly oblong, divergent dorsally, slightly closer to each other than to the eye; dorsal margin weakly convex but not attaining dorsal margin of eye, which is elevated (Figs 19, 23); lateral margin below eyes straight, slightly upturned; frontoclypeus acute, sutures vertical, joining horizontally (truncate dorsally). ***Pronotum*.** Longitudinally divided into 2 parts by strong dorsal constriction (Figs 18, 22), anterior part elevated, evenly convex with pair of slender suprahumeral spines directed laterally (Figs 18, 19, 22, 23), posterior part swollen and bearing 3 apical, slender spines (Figs 18, 22). ***Wings*.** Forewing with 1 basal cell (M) (Fig. 24), 1 discoidal cell (R_2+3_) (Fig. 21), basal cell formed by divergent M and Cu at base, then convergent and completely fused into single vein, then separate again distally (1^st^ m-cu crossvein absent). Hind wing with veins R and M briefly confluent, and 1 m-cu crossvein, with forked anal vein (Fig. 21). ***Legs*.** Metathoracic tibia with cucullate setal row I double, row II and row III complete and single. Male similar to female.

##### Distribution.

Neotropical: South America.

##### Etymology.

The name is feminine and refers to the five (*quinque*-) spines (-*spinosa*) on the pronotum.

**Figures 25–31.**
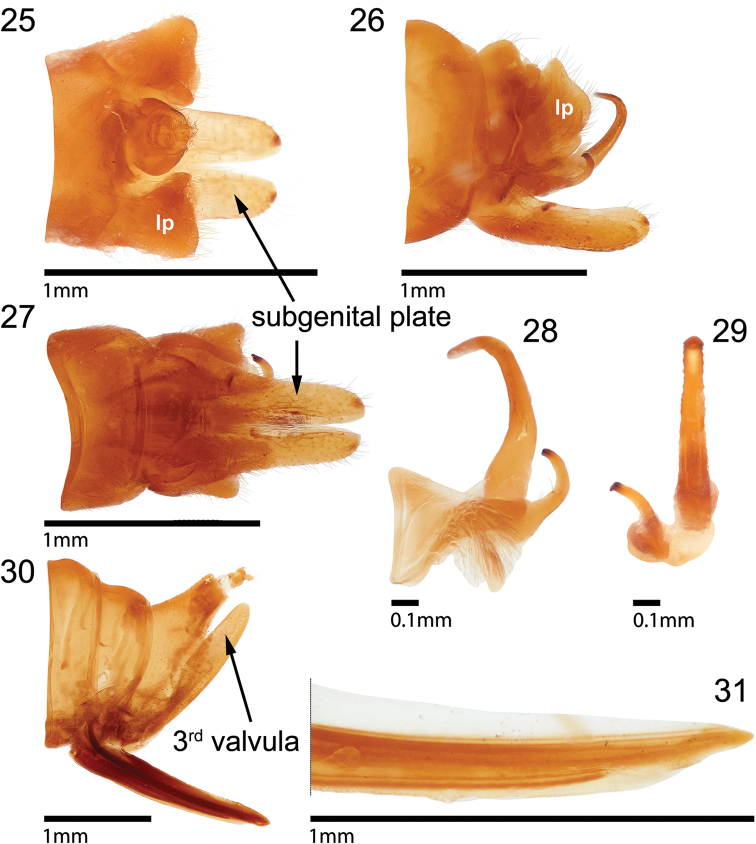
Terminalia of *Quinquespinosaseptamacula* sp. nov. **25** male pygofer and subgenital plate in dorsal view **26** undissected male terminalia **27** male pygofer and subgenital plate in ventral view **28, 29** aedeagus and left style in lateral and posterior views, respectively **30** undissected female terminalia **31** distal half of second valvula, lateral view. *lp*, lateral plate.

##### Notes.

Whereas *Euritea* has two m-cu crossveins in the forewing, this new genus has only one. Its veins M and Cu separate and diverge at base, then instead of being bridged with an m-cu crossvein as in *Euritea* and *Ceresinoidea*, its veins M and Cu completely fuse into a single vein (enclosing basal cell M; Figs 21, 24), then separate again distally as in all other Smiliinae. This unusual venation at the wing base is the same on all wings of all 17 specimens, so is not an aberration. Another interesting feature of this genus is the confluence with the anterior branch of R with M for a short distance, in the hind wing (Fig. 21); this trait also occurs in Ceresini as well as in other Smiliinae.

#### ﻿ Quinquespinosa septamacula sp. nov.

Taxon classificationAnimaliaHemipteraMembracidae

16E66E40-3225-5829-B8A8-D1E37AEDD128

https://zoobank.org/7DA19879-CE17-4901-9280-B6BF0130266E

Figs 18–24 , 25–31

##### Diagnosis.

Frontoclypeal sutures bordered by conspicuous black spots; pronotum with pair of dorsal pale longitudinal stripes dorsally and another pair more laterally, at level of suprahumeral spines; posterior portion of pronotum with 7 distinct dark marks (Figs 22, 24): 2 pairs, one pair straddling the apical middle spine and the second pair more laterally, behind the bases of apical lateral spines, and one on each of the 3 posterior spines.

##### Description.

Measurements (mm). Length with forewing in repose ♂ 7.0–7.5, ♀ 8.0–8.5; width across suprahumeral spines ♂ 3.6–4.0, ♀ 4.1–4.3; height in anterior view ♂ 3.1–3.2, ♀ 3.2–3.4. ***Pronotum*.** With apical lateral spine extending to Cu vein, middle spine attaining mid-point of Cu and M _3+4_ (Fig. 20). ***Terminalia*.** Male. Pygofer with lateral plate large, subquadrate in dorsal view (Fig. 25); subgenital plates subtriangular, tips rounded (Fig. 27); style recurved with acute apex (Figs 27–29); aedeagus narrow throughout, gradually recurved, its sides bearing short sharp points along outer margin (Figs 26, 28, 29). Female first valvula gradually narrowed, dorsal margin smooth in basal 3/4^ths^, weakly crenulate in distal ¼ (Fig. 30); second valvula dorsally smooth throughout (Fig. 31). ***Color*** (female Figs 18–20, male Figs 22–24). Yellow orange throughout except with 2 black marks along head lateral margin and pair straddling frontoclypeus larger than pair just below eyes, and in traverse narrow band on eyes (Figs 19, 23). Pronotum surprahumeral spine apex black, 4–5 pale longitudinal stripes (along lateral margins and laterally) in both genders (Figs 18–20, 22–24), and in male (Fig. 22) also on medial carina, and 7 black marks posteriorly (Figs 22, 24): 1 pair straddling base of middle apical spine, 1 pair lateral behind base of each lateral spine, and one on apex of each apical spine.

##### Material examined.

***Holotype*** ♂ (EPNC) with labels “ECUADOR: NAPO: Reserva Ethnica | Waorani, 1 km. S Onkone Gare | Camp Trans. Ent 9. Feb 1995 | 220m | 11-Feb-1995 00 °39'10"S 076 °26'W | T.L Erwin: et al “, “Insecticidal fogging of mostly bare | green leaves, some with covering | of lichenous or bryophytic plants in | terre firme forest At Trans 1, | Sta. 2 Project MAXUS Lot 1021.” and red “HOLOTYPE | Quinquespinosa | septamacula | S.H. McKamey.” ***Non-types***: 16 specimens. Two (USNM) have the same data as the holotype except as noted: 1♀ 8-Feb-1996 Lot 971; 1♀ 29-Jun-1994 lot 755. The other specimens have the same data as the holotype except coordinates 00 °39'25.7"S 076 °27'10.8"W and otherwise noted: 1♀ 8-Feb-1996 lot#1470 (EPNC); 1♂, 1♀, 8-Feb-95 Lot 952 (USNM); 1♂ 6-Oct-1994 Lot 873 (EPNC); 1♀ 15-Jan-1994 Lot 579 (USNM); 2♂ 6-Jul-1995 Lot 1114 (EPNC); 1♀ 7 Oct-1995 Lot 1239 (EPNC); 1♀, 8-Feb-1996 Lot 1469 (USNM); 1♀ 3-Oct-1996 Lot 1729 (USNM). There are four non-Ecuadorian specimens: 1♂, (INPA) “BRAZIL: AMAZONAS | Rio Januaca, 40 | km sw Manaus | 10 Mar 1979 | 03 °20' S. 060 °17'W”, “Montgomery, Erwin, | Sucharaov, Scxhimmel. | Kirischik, Date, | Bacon, Collectors”, “White water inun- | dation forest canopy | fogged with Pyrethrum | Sample #62. 1♀ (MNHN) “CAMOPI-OYAPOCK | GUYANE 19.Nov-1969, “GUYANE MISSION | BALACHOWSKY-GRUNER | OCT-NOV.1969”, “Piege | lumineux”, “Muséum Paris | 1095-5”. 2♀ (1 MUSM, 1 USNM) with labels “PERU: MADRE DE DIOS | Rio Manu, BIOLAT Biol. Sta., | Pakitza, 356m 26 Sep 1991 | 11°56'47"S 071°17'00"W” | T.L. Erwin”, “Insecticidal fog of bamboo at 4m | green, scattered dry leaves, stems | Tr. Zungaro /3.5 Lot 121.”

##### Distribution.

Brazil, Ecuador, French Guiana, Peru.

##### Etymology.

The specific epithet is feminine, based on the seven (*septa*-) black marks (-*macula*) on the posterior portion of the pronotum.

##### Notes.

There is variability in the length of the suprahumeral spines and the size of the four preapical black spots (compare Figs 18 and 22); neither is correlated to body size or gender. The pronotum of the specimen from French Guiana (MNHN) is unique in being black only on the tips of the five spines, lacking the four other black marks altogether and is considered to be a variety, possibly geographical, of the same species.

The 13 specimens from Ecuador fogging samples in the Reserva Etnica Waorani were collected in January, February, June, July, and October, from 1994–1996. The Peruvian and French Guiana specimens were collected in September (1991) and November (1969), and the Brazilian specimen in March (1979). Considered together, the only gaps are April, May, August, and December. The April-May gap possibly represents the growth of a second generation but the one-month gaps are probably too short to indicate other generations. Other explanations are sampling error, annual or seasonal fluctuations in climate, or that the adults are present throughout the year at least somewhere in their large range.

All specimens were collected by insecticidal fogging of the tree canopy (one from inundation forest and the others from terre firme forest) except the specimen from French Guiana, which was collected at a light trap. Although various leafhoppers feed on bamboo, no treehoppers have been found feeding on it, so the bamboo record for the Peruvian specimen is probably not its host plant.

#### ﻿ Tectiforma gen. nov.

Taxon classificationAnimaliaHemipteraMembracidae

B4358F86-4B2C-5927-983D-01AED53209F7

https://zoobank.org/8CCAFDF0-B705-4A10-A6F1-FC7DAB17E8DB

Figs 32–38 , 39

##### Type species.

*Tectiformaguayasensis* sp. nov.

##### Diagnosis.

This is the only acutaline genus with the pronotum tectiform throughout.

##### Description.

**Figures 32–38.**
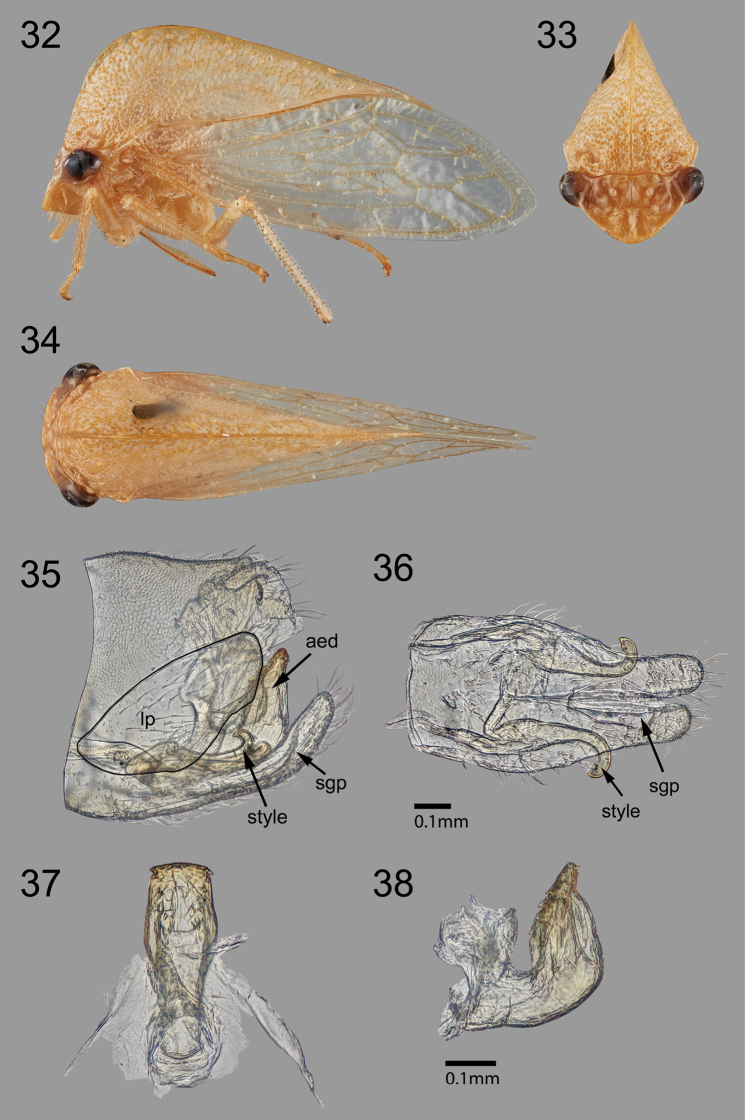
*Tectiformaguayasensis*, sp. nov. holotype **32–34** habitus view in lateral, anterior, and dorsal views, respectively **35** undissected pygofer and genitalia **36** subgenital plate and styles, ventral view **37–38** aedeagus in anterior and lateral views, respectively. *aed*, aedeagus; *lp*, lateral plate; *sgp*, subgenital plate.

**Figure 39.**
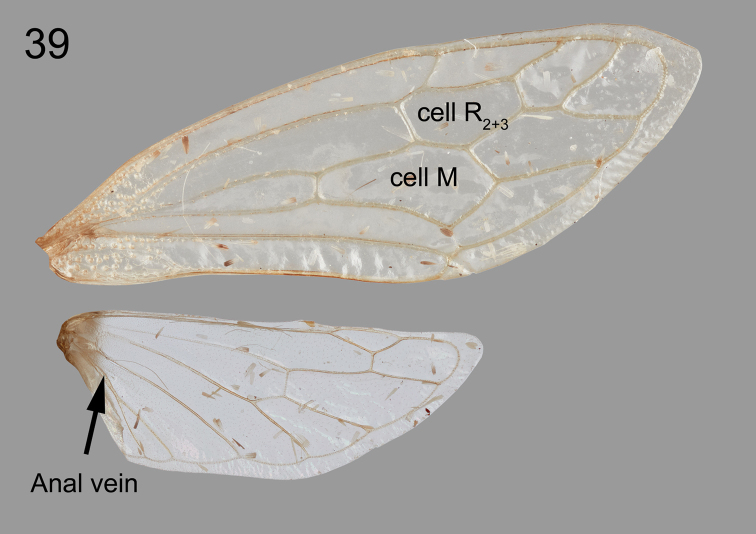
*Tectiformaguayasensis* sp. nov. holotype, wings, showing the two discoidal cells in the forewing and the forked anal vein in the hind wing.

Overall body slender (Fig. 34). ***Head*.** Vertex inclined slightly forward, aligned with steep pronotal metopidium (Fig. 32); head vertex uneven, slightly swollen just ventrolateral of ocellus, glabrous, dorsal margin weakly sinuate, not attaining dorsal margin of eye, which is elevated (Fig. 33), ventral margin including frontoclypeus evenly convex ventrally with and convex, narrow vertical carina, its sutures evenly arched to middle; ocelli slightly oblong, divergent dorsally, slightly closer to each other than from eye (Fig. 33). ***Pronotum*.** Elevated anteriorly (Fig. 32), lacking suprahumeral spines (Figs 33, 34), laterally compressed and strongly tectiform from top of metopidium and posteriorly (Figs 32, 33); metopidium in lateral view steeply inclined, gradually convex, then descending in straight line to apex; apex extends to mid-point between veins Cu and M_3+4_ (Fig. 32). ***Wings*.** Forewing (Fig. 39, top) with 2 adjacent discoidal cells (R_2+3_ and M), 2 m-cu crossveins. Hind wing (Fig. 39, bottom) with 1 r-m and 1 m-cu crossvein, with forked anal vein. ***Legs*.** Metathoracic tibia with cucullate setae row I double, row II and row III complete and single.

##### Distribution.

Neotropical.

##### Etymology.

The name is feminine and based on the strongly tectiform pronotum.

##### Notes.

The forewing venation, with two discoidal cells, is almost identical to that of *Euritea*, the only difference being that in *Euritea*, the two discoidal cells are not adjacent to each other. The dorsomedial carina of *Cornutalisandinum*Flórez-V (2017) and *Ceresinoideazacki* are tectiform, but in the new genus *Tectiforma* the entire pronotum is tectiform, attaining a much greater height above the humeral angle, so these cannot be confused for one another even without considering differences in forewing venation.

#### ﻿ Tectiforma guayasensis sp. nov.

Taxon classificationAnimaliaHemipteraMembracidae

5A165518-3D42-5B5F-80D2-332DF466BA1F

https://zoobank.org/241E811B-A3B3-4CEC-B68B-579231660AFC

Figs 32–38 , 39

##### Diagnosis.

Same as for genus: slender, pale green, with pronotum strongly tectiform.

##### Description of male.

Measurements (mm). Length with forewing in repose 6.7; width across humeral angles 2.2; height in anterior view 2.9. ***Pronotum*.** As described for genus. ***Terminalia*.** Pygofer including lateral plate subquadrate in lateral view (Fig. 35); lateral plate large, ovoid, unarmed, bearing setae; styles (Figs 35, 36) simple, distally recurved and acute; aedeagus U-shaped in lateral view (Fig. 38), shaft apex with two small posterior spines (Fig. 38), anterior surface with 2 columns of 5 larger spines each, inset from swollen lateral margins (most visible in lateral view, Fig. 38). ***Color*.** Pale green throughout.

**Female** unknown.

##### Material examined.

***Holotype*** ♂ (USNM) with labels “ECUADOR: Guayas. | Hac. San Joaquin, | 4 rd km SW Bucay | 1–4 May 1986 250m.”, “S.H. McKamey lot | #86-0501-UV”, and a red “HOLOTYPE | Tectiforma | guayasensis | S.H. McKamey”.

##### Distribution.

Ecuador.

##### Etymology.

The specific epithet is based on Guayas, the province in which the holotype was collected.

##### Note.

Collected at an ultraviolet light.

## ﻿Conclusions

Some of the above new species, most notably *Ceresinoideazacki* and *Quinquespinosaseptamacula*, are superficially similar to members of the tribe Ceresini. The distinctly tectiform posterior pronotom of *C.zacki* resembles some inornate Ceresini. In contrast, most inornate ceresine males have a slender lateral plate that bears a short to long protruding process, or “lateral tooth” (Kopp and Yonke 1979). *Quinquespinosaseptamacula* resembles some ornate Ceresini. Most ornate ceresine males have lateral plates unarmed, as in *Q.septamacula*. However, all the above new species have the two sides of the male subgenital plate deeply divided (Figs 13, 27, 36), in contrast to Ceresini (Kopp and Yonke 1979). Additionally, all the new taxa described here have forewing venation consistent with Acutalini, not Ceresini: basally separated R, M, and Cu veins, as opposed to Ceresini species, which have the forewing veins R and M completely fused basally and strongly divergent for a short distance near the middle of the wing.

## Supplementary Material

XML Treatment for Ceresinoidea

XML Treatment for Ceresinoidea zacki

XML Treatment for Quinquespinosa

XML Treatment for Quinquespinosa septamacula

XML Treatment for Tectiforma

XML Treatment for Tectiforma guayasensis
